# Protection of rat skeletal muscle fibers by either L-carnitine or coenzyme Q10 against statins toxicity mediated by mitochondrial reactive oxygen generation

**DOI:** 10.3389/fphys.2013.00103

**Published:** 2013-05-15

**Authors:** P. G. La Guardia, L. C. Alberici, F. G. Ravagnani, R. R. Catharino, A. E. Vercesi

**Affiliations:** ^1^Departamento de Patologia Clínica, Faculdade de Ciências Médicas, Universidade Estadual de CampinasCampinas, Brazil; ^2^Innovare Biomarkers Laboratory, Faculdade de Ciências Médicas, Universidade Estadual de CampinasCampinas, Brazil

**Keywords:** statins, skeletal muscle mitochondrial dysfunction, myotoxicity, L-carnitine, coenzyme Q10

## Abstract

Mitochondrial redox imbalance has been implicated in mechanisms of aging, various degenerative diseases and drug-induced toxicity. Statins are safe and well-tolerated therapeutic drugs that occasionally induce myotoxicity such as myopathy and rhabdomyolysis. Previous studies indicate that myotoxicity caused by statins may be linked to impairment of mitochondrial functions. Here, we report that 1-h incubation of permeabilized rat soleus muscle fiber biopsies with increasing concentrations of simvastatin (1–40 μM) slowed the rates of ADP-or FCCP-stimulated respiration supported by glutamate/malate in a dose-dependent manner, but caused no changes in resting respiration rates. Simvastatin (1 μM) also inhibited the ADP-stimulated mitochondrial respiration supported by succinate by 24% but not by TMPD/ascorbate. Compatible with inhibition of respiration, 1 μM simvastatin stimulated lactate release from soleus muscle samples by 26%. Co-incubation of muscle samples with 1 mM L-carnitine, 100 μM mevalonate or 10 μM coenzyme Q10 (Co-Q10) abolished simvastatin effects on both mitochondrial glutamate/malate-supported respiration and lactate release. Simvastatin (1 μM) also caused a 2-fold increase in the rate of hydrogen peroxide generation and a decrease in Co-Q10 content by 44%. Mevalonate, Co-Q10 or L-carnitine protected against stimulation of hydrogen peroxide generation but only mevalonate prevented the decrease in Co-Q10 content. Thus, independently of Co-Q10 levels, L-carnitine prevented the toxic effects of simvastatin. This suggests that mitochondrial respiratory dysfunction induced by simvastatin, is associated with increased generation of superoxide, at the levels of complexes-I and II of the respiratory chain. In all cases the damage to these complexes, presumably at the level of 4Fe-4S clusters, is prevented by L-carnitine.

## Introduction

Statins are natural (fungal-derived) or synthetic inhibitors of the enzyme HMG-CoA reductase that catalyzes the conversion of 3-hydroxy-3-methylglutaryl coenzyme-A (HMG-CoA) into mevalonic acid, the rate-limiting step in cholesterol synthesis (Endo, 1992). High plasma levels of cholesterol are well established as independent risk factor for coronary heart disease (Sirvent et al., 2005a) that can be significantly reduced by statins treatment (Tobert et al., 1982). The efficacy and tolerability of statins are well documented (Tobert et al., 1982; Sirvent et al., 2005b). Although this treatment normally lowers morbidity and mortality (LIPID Study Group, 1998) about 10% of the patients (Bruckert et al., 2005) develop myophatic symptoms and approximately one in 7.5 million patients develop fatal rhabdomyolysis (Venero and Thompson, 2009). Myopathic symptoms caused by statins, such as muscle cramps, myalgia, weakness and exercise intolerance can occur with or without increase in plasma creatine kinase levels (Sirvent et al., 2005a).

Statins block cholesterol synthesis early in its metabolic pathway therefore they also decrease the production of both ubiquinone (Co-Q10) and other important metabolites including dolichols and other prenylated isoprenoids required for muscle cell functions (Sirvent et al., 2008). Co-Q10 is an important respiratory chain electron transporter that also displays antioxidant properties in its reduced form (ubiquinol). Although the molecular mechanisms underlying statins induced myotoxicity are not well established the most popular hypothesis proposes that it is mediated by inhibition of mitochondrial respiration as a consequence of Co-Q10 depletion (Ghirlanda et al., 1993; Laaksonen et al., 1995; Thibault et al., 1996; Miyake et al., 1999; Rundek et al., 2004; Paiva et al., 2005; Littarru and Langsjoen, 2007; Mabuchi et al., 2007; Young et al., 2011; Bookstaver et al., 2012). In addition, several studies using isolated mitochondria or intact cells propose that statins promote cell death mediated by mitochondrial dysfunctions associated with alterations in calcium homeostasis, inhibition of beta-oxidation, inhibition of complex I of the electron transport chain and mitochondrial oxidative stress (Sirvent et al., 2005b; Yasuda et al., 2005; Kaufmann et al., 2006; Velho et al., 2006; Oliveira et al., 2008; Skottheim et al., 2008; Itagaki et al., 2009; Kwak et al., 2012).

Data from our group (Velho et al., 2006) showed that statins stimulate Ca^2+^ induced mitochondrial permeability transition (MPT) *in vitro*, in mitochondria isolated from control mice liver or in liver mitochondria isolated from mice treated with lovastatin (100 mg/kg daily via gavage, during 15 days). In addition, Sacher et al. (2005) reported that simvastatin or lovastatin (1–100 μM) activate the mitochondrial pathway of apoptosis in primary human skeletal muscle cells obtained from skeletal muscle biopsies of healthy individuals. With respect to the mechanisms of cell death induced by statins, we have previously shown that, at low concentrations (≤10 μM), simvastatin induces apoptosis in PC3 prostate cancer cells. At these low concentrations mevalonate but not cyclosporine A, an inhibitor of MPT, prevented cell death (Oliveira et al., 2008). At higher concentrations (≥60 μM) simvastatin-induced necrosis was sensitive to cyclosporine A but not to mevalonate, indicating that, at high concentrations, the toxicity of statins is not solely the result of HMG-CoA reductase inhibition. In addition, cell necrosis was preceded by a threefold increase in the concentration of cytosolic free Ca^2+^ and MPT (Oliveira et al., 2008). More recently Costa et al. (2013) provided evidence that simvastatin-induced MPT and cell necrosis were inhibited by L-carnitine and piracetam in a dose-dependent fashion; when combined, L-carnitine and piracetam acted at concentrations significantly lower than they act individually. These results shed new light into both the cytotoxic mechanisms of high statins concentrations and the mechanisms underlying the protection against MPT and cell death by the compounds L-carnitine and piracetam (Costa et al., 2013).

L-carnitine, beyond the physiological functions on fatty acids transport across the inner mitochondrial membrane, has the properties to scavenge reactive oxygen (Gulcin, 2006; Mescka et al., 2011) and to bind Fe^2+^ (Gulcin, 2006) a transition metal, supposed to participate in the mitochondrial oxidative stress that leads to MPT (Castilho et al., 1995). Therefore, the aims of this study were double: first, to analyze the events leading to simvastatin induced skeletal muscle toxicity, at low concentrations (1 μM), and second, to better understand the mechanism underlying mitochondrial protection against reactive oxygen by L-carnitine or Co-Q10.

The results presented here indicate that simvastatin induced inhibition of respiration is mediated by the attack of mitochondrially generated superoxide radicals to the respiratory chain complexes I and II probably at the level of 4Fe-4S clusters. In addition, both L-carnitine and Co-Q10 act directly as radical scavenger in the protection against simvastatin-induced oxidative damage to skeletal muscle mitochondria.

## Materials and methods

### Chemicals and reagents

For all experiments, the reagents used were of analytical grade. Adenosine 5′-diphosphate monopotassium salt dihydrate (ADP), adenosine 5′-triphosphate monopotassium salt dihydrate (ATP), bovine serum albumin (BSA), dimethyl sulfoxide (DMSO), ethylene-bis(oxyethylenenitrilo)tetraacetic acid (EGTA), oligomycin, L-glutamic acid, L-malic acid, carbonyl cyanide 4-(trifluoromethoxy) phenylhydrazone (FCCP), 4-(2-hydroxyethyl)piperazine-1-ethanesulfonic acid (HEPES) Phospho-creatine, taurine, (2-[N-Morpholino] ethanesulfonic acid) monohydrate, ascorbic acid, oxaloacetic acid, imidazole, K-lactobionate, simvastatin, ubiquinone, β-Nicotinadenineamine dinucleotide were obtained from Sigma-Aldrich (St. Louis, MO, USA). The ADP, glutamate and malate solutions were prepared by dissolving the acids in water and adjusting the pH to 7.2 with KOH.

### Animals

Wistar female rats with 10–12 weeks of age had access to standard laboratory rodent chow diet and water *ad libitum* and were housed at 22 ± 2°C on a 12 h light-dark cycle. The experiments were approved by the Committee for Ethics in Animal Experimentation at the university and are in accordance with the Guide for the Care and Use of Laboratory Animals published by the National Academy of Sciences.

### Skeletal muscle sample preparation

Soleus muscle tissues were harvested from rats and placed in ice-cold relaxing solution [containing 10 mM Ca-EGTA buffer (2.77 mM of CaK_2_EGTA + 7.23 mM K_2_EGTA) free concentration of calcium 0.1 mmol/L, 20 mmol/L imidazole, 50 mmol/L K^+^/4-morpholinoethanesulfonic acid, 0.5 mmol/L dithiothreitol, 7 mmol/L MgCl_2_, 5 mmol/L ATP, 15 mmol/L phosphocreatine, pH 7.1]. Two to three milligram of soleus skeletal muscle were utilized and individual fiber bundles were separated with 2 forceps. Samples were permeabilized for 30 min in ice-cold relaxing solution with saponin (50 μg/mL) gently stirred and washed 3 times with MiR05 medium (60 mmol/L potassium lactobionate, 0.5 mmol/L EGTA, 3 mmol/L MgCl_2_, 20 mmol/L taurine, 10 mmol/L KH_2_PO_4_, 20 mmol/L HEPES, 110 mmol/L sucrose, 1 g/L BSA, pH 7.1) at 4°C. Samples were dried with filter paper and weighted (Kuznetsov et al., 2008).

### Oxygen consumption measurements

Oxygen consumption in permeabilized skeletal muscle tissues was measured in a medium MiR05 at 37°C, in the presence of 10 mM glutamate and 5 mM malate, or 5 mM succinate, or 50 μM TMPD plus 2 mM ascorbate plus 1 μM antimycin A using a high resolution oxygraph OROBOROS (Innsbruck, Austria). Simvastatin or Dimetilsulfóxido (DMSO) was incubated by 1 h. 400 μM ADP, 1 μg/mL oligomycin, and 0.2 μM FCCP were added during experiments (Kuznetsov et al., 2008).

### Citrate synthase (CS) activity

The conversion of oxaloacetate and acetyl-CoA to citrate and SH-CoA catalyzed by citrate synthase was monitored by measuring the colorimetric product thionitrobenzoic acid (Shepherd and Garland, 1969). Soleus skeletal muscle homogenates (0.5–0.75 mg/mL, wet weight) were incubated at 30°C in a buffer containing 50 mM tris-HCl (pH 8.0), 0.1% Triton X-100, 250 μM oxaloacetate, 50 μM acetyl-CoA, and 100 μM 5,5′-dithiobis(2-nitrobenzoic acid). The increase in absorbance at 412 nm was monitored for 6 min using a microplate reader (Power Wave XS 2, BioTek Instruments, Winooski, VT, USA).

### Lactate assay

Lactate production was monitored by means of changes in NADH fluorescence. Medium containing 50 mM hydroxylamine, 50 mM tris, pH10.0, 800 μM NAD^+^, 40 U lactate dehydrogenase and an aliquot of medium MiR05 containing the non-permeabilized sample prior incubation with simvastatin or DMSO for 1 h, at 25°C. Calibration was made by addition of know concentrations of lactate. A Hitachi F4500 spectrofluorometer operating at excitation and emission wavelengths of 366 and 450 nm, respectively, was used to measure the changes in NADH fluorescence.

### Hydrogen peroxide release

Soleus skeletal muscle samples (~20 mg) were pre incubated with simvastatin or DMSO for 1 h in medium MiR05 plus 10 μM Amplex red (Molecular Probes, Invitrogen, Carlsbad, CA) and 1 U/mL horseradish peroxidase. Calibration was made by addition of known concentrations of hydrogen peroxide. Changes in fluorescence were monitored using a spectrofluorometer (Hitachi F4500) operated at excitation and emission wavelengths of 563 and 587 nm, respectively (Anderson and Neufer, 2006).

### Samples for coenzyme Q10 assay

Standard coenzyme Q10 (≥98%purity) was purchased from Sigma-Aldrich (USA). HPLC-grade water was prepared using a MilliQTM System (Millipore Corporation). Methanol HPLC-grade was purchase from Merck Chemicals (Germany), Ethanol analytical grade and perchloric acid were purchased from F. MAIA (Brazil). Benzene (light petroleum) was purchased from VETEC (Brazil). Samples from soleus skeletal muscle (around 100 mg) from Wistar rats were prepared as previously described by Redfearn and Whittaker (1966) with modifications (Redfearn and Whittaker, 1966). Briefly, muscle tissue samples were homogenized in medium containing 1 mL of MiR05 medium, 1 mL of 0.6 M perchloric acid, and 3 mL of cold methanol. The homogenates were vortexed during 30 s, 5 mL of benzene were added and the samples were vortexed again for 30 s. The samples were centrifuged at 5000× g for 10 min (room temperature). The upper phase was collected and dried under nitrogen flux. The precipitate obtained was dissolved in 50 μL of hexane.

### HPLC assay for coenzyme Q

Coenzyme Q10 was determined using a chromatographic system Shimadzu LC (Japan) in conjunction with a SPD-10A UV-Visible set to 275 nm. Twenty microliters of the samples in hexane were injected into the analytical column (Luna 250 × 4.6 mm; C18 (2) 100A; 5 μm particle size; Phenomenex®) maintained at 25°C. Coenzyme Q10 was eluted from the column at a flow rate of 1.4 mL/min using a isocratic mode linear gradient of methanol:ethanol (65:35). Lower and upper limits of detection for total coenzyme Q10 were confirmed at the following concentrations: 0.167 μmol/L and 150 μmol/L. The lower and upper limits of linearity were observed at the following concentrations: 0.5 μmol/L and 50.0 μmol/L.

### Statistical analyses

The results of experiments performed in at least five independent experiments are displayed as means ± S.E.D and significance was assessed by ANOVA, followed by the Tukey post-test, or student-*t* test with significance level set at *p* < 0.05 using Sigma Stat 3.1 (Systat, San Jose, CA, USA).

## Results

### Inhibition by simvastatin of ADP- or FCCP-stimulated oxygen consumption supported by complex I substrates in skeletal muscle fibers

**Figure 1:**
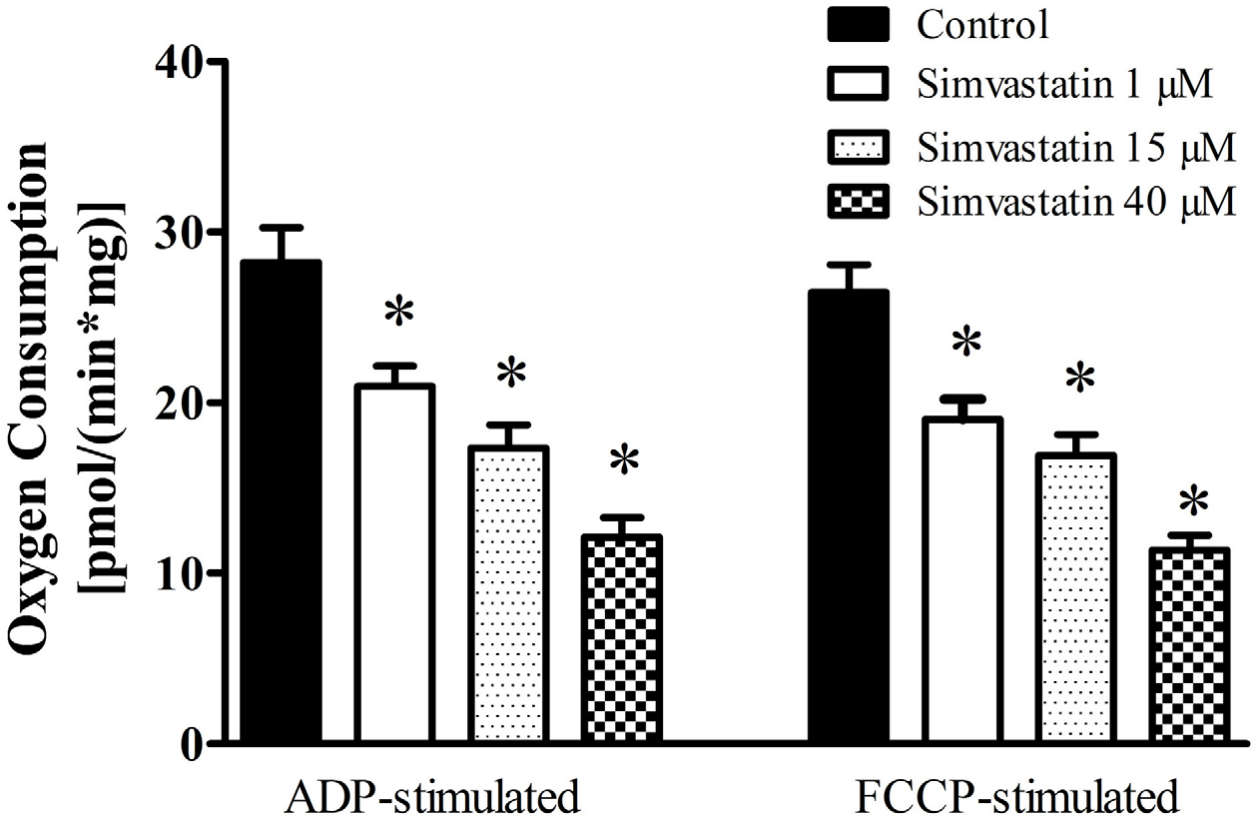
**Inhibition by simvastatin of ADP- or FCCP-stimulated oxygen consumption supported by complex I substrates in skeletal muscle.** Skeletal muscle samples were incubated in MiR05 medium in the presence of 1, 15, and 40 μM simvastatin, or DMSO for 1 h. ADP (400 μM) or 1 μg/mL oligomycin plus 0.2 μM FCCP were added where indicated. Respiration was supported by 10 mM glutamate plus 5 mM malate. ^*^p < 0.05 vs. control by one-way analysis of variance. *N* = at least 6 independent experiments.

In order to investigate the effects of simvastatin on soleus skeletal muscle mitochondrial respiration “*in vitro*” we incubated the permeabilized bundles for 1 h in the standard incubation medium (MiR05) containing increasing concentrations (1, 15, or 40 μM) of simvastatin or 0.1% DMSO as control. Oxygen consumption supported by 10 mM glutamate plus 5 mM malate was monitored before and after the sequential additions of 400 μM ADP, 1 μg/mL oligomycin, and 0.2 μM FCCP. Figure 1 shows that simvastatin promoted a dose dependent inhibition of ADP- and FCCP-stimulated respiration but did not affect the rate of resting respiration (data not shown). The inhibition caused by 1 μM simvastatin was 25 and 27% for ADP- and FCCP-stimulated respiration, respectively. The inhibition peaked at 57% in the presence of 40 μM simvastatin for both ADP- and FCCP-stimulated respiration.

Considering that typically prescribed daily oral doses of statins 20–80 mg (Kwak et al., 2012) generate concentration peaks in skeletal muscle in the range of 2–5 μM, we choose the concentration of 1 μM simvastatin to perform the next experiments.

### Inhibition by simvastatin of ADP-stimulated oxygen consumption supported by succinate

**Figure 2:**
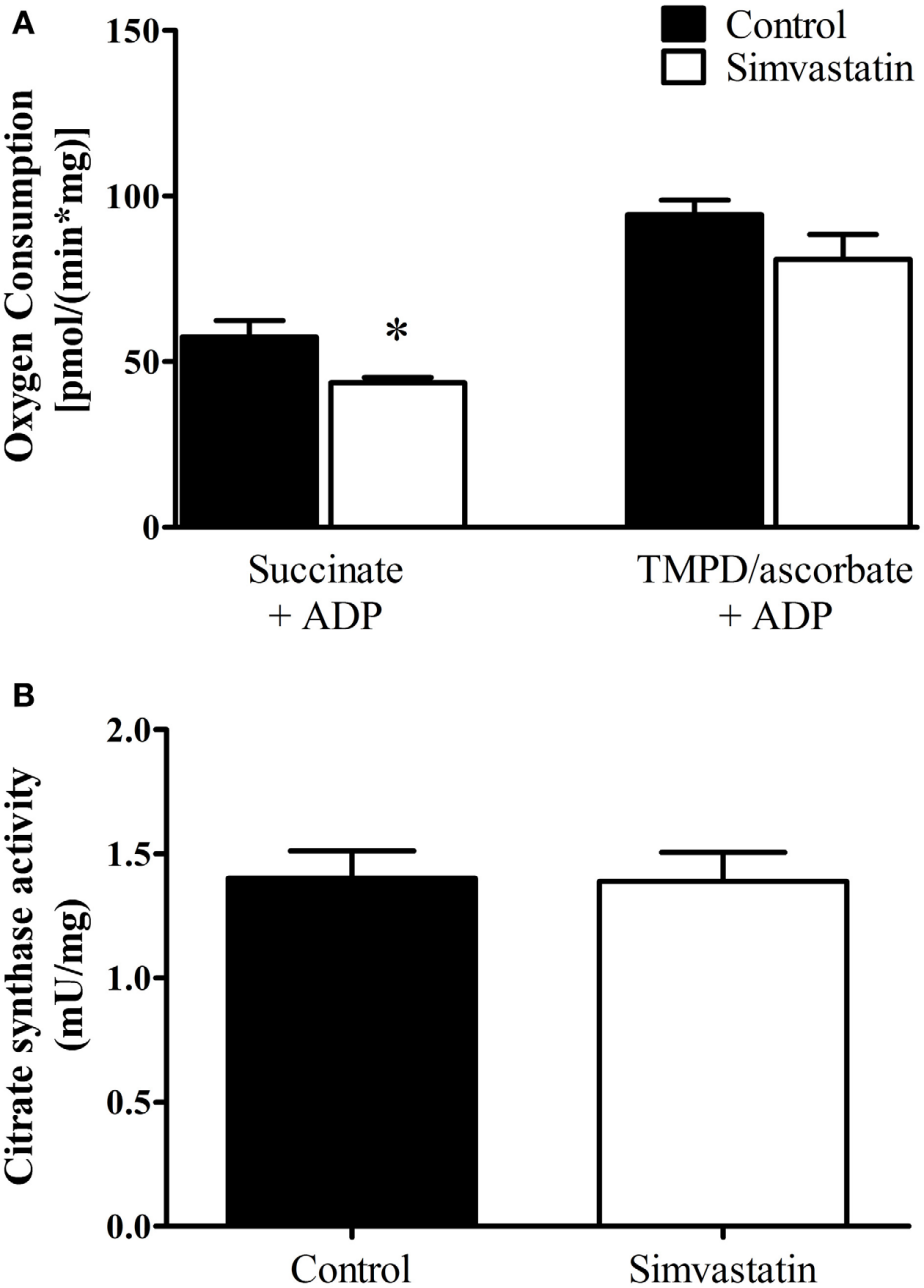
**Inhibition by 1 μM simvastatin of ADP-stimulated oxygen consumption supported by succinate but not by TMPD/ascorbate. (A)** Skeletal muscle samples were incubated in MiR05 medium in the presence of 1 μM simvastatin, or DMSO for 1 h. ADP (400 μM) or 1 μg/mL oligomycin plus 0.2 μM FCCP were respectively added. The respiration was supported by 5 mM succinate or 5 μM TMPD plus 2 mM ascorbate plus 1 μM antimycin A. **(B)** Skeletal muscle homogenates were incubated at 30°C in a buffer containing 50 mM tris-HCl (pH 8.0), 0.1% Triton X-100, 250 μM oxaloacetate, 50 μM acetyl-CoA, and 100 μM 5, 5′-dithiobis(2-nitrobenzoic acid) to assay citrate synthase activity. Prior preparation of homogenates, skeletal muscle samples were incubated by 1 h in MiR05 medium in presence of 1 μM simvastatin. ^*^p < 0.05 vs. control by student *t*-test. *N* = at least 5 independent experiments.

The results on respiratory complex II inhibition by statins reported in the literature are controversial (Sirvent et al., 2005a, 2012; Bouitbir et al., 2012a). Here we analyzed the effect of 1 μM simvastatin on succinate or TMPD/ascorbate supported respiration. One hour incubation of the skeletal muscle preparation with 1 μM simvastatin caused 24% inhibition of ADP-stimulated respiration supported by succinate but did not significantly change the rate of TMPD/ascorbate supported respiration (Figure 2A). In addition, the citrate synthase activity assay applied to skeletal muscle tissue incubated during 1 h in the presence of 1 μM simvastatin indicated that mitochondrial density and number were not changed by the statin treatment (Figure 2B).

### Inhibition by 1 μM simvastatin of ADP- or FCCP-stimulated oxygen consumption supported by complex I substrates was prevented by mevalonate, Co-Q10 or L-carnitine

**Figure 3:**
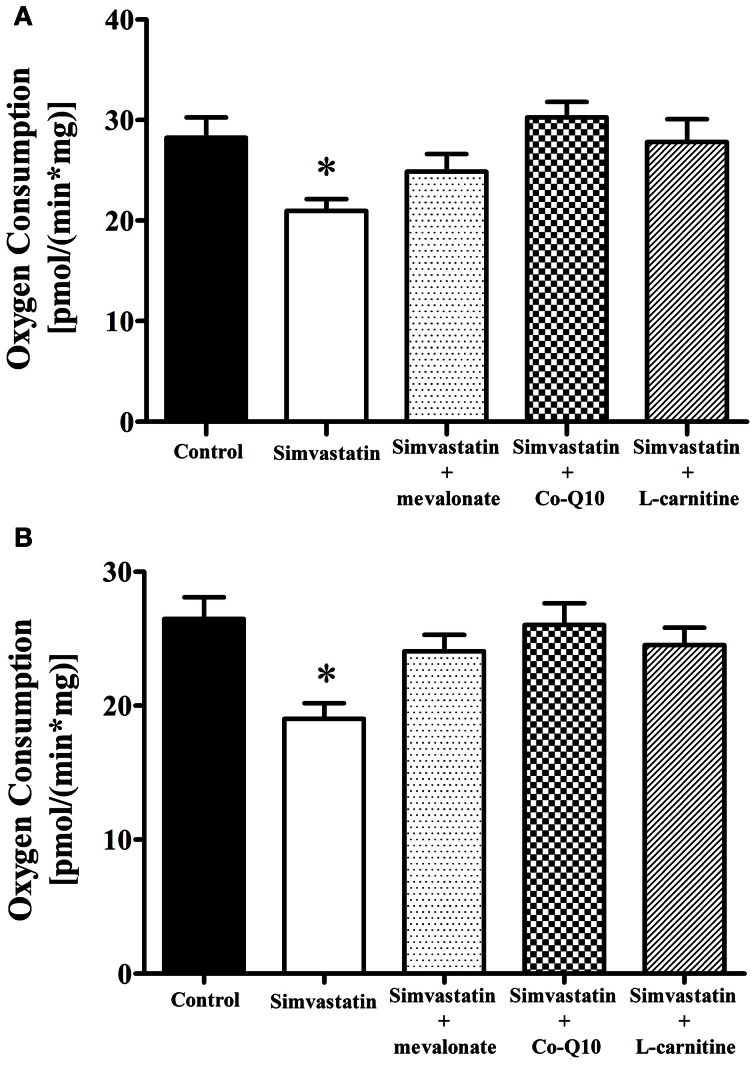
**Inhibition by 1 μM simvastatin of (A) ADP or (B) FCCP-stimulated oxygen consumption supported by complex I substrates was prevented by mevalonate, Co-Q10 or L-carnitine.** Skeletal muscle samples were incubated in MiR05 medium in the presence of 1 μM simvastatin, or DMSO for 1 h containing 100 μM mevalonate, 10 μM coenzyme Q10, or 1 mM L-carnitine. ADP (400 μM) or 1 μg/mL oligomycin plus 0.2 μM FCCP were added and oxygen consumption was supported by 10 mM glutamate plus 5 mM malate. Since the control and 1 μM simvastatin bars were not statically different in Figure 1 and panel **(A)**, we used a general averages for controls and 1 μM simvastatin in both figures. ^*^p < 0.05 vs control by one-way analysis of variance. *N* = at least 6 independent experiments.

The first step to elucidate the mechanism of simvastatin induced skeletal muscle respiration inhibition was the co-incubation with mevalonate, the product of the reaction catalyzed by the enzyme HMG-CoA reductase. Figures 3A,B show that 100 μM mevalonate protected against simvastatin-induced inhibition of complex I substrates supported respiration stimulated by ADP or FCCP, respectively. The next step was the co-incubation with coenzyme Q10, another product of cholesterol *de novo* biosynthesis. It can be seen that 10 μM Co-Q10 similarly to mevalonate significantly protected against the inhibition of complex I respiration stimulated by ADP- or FCCP (Figures 3A,B). L-carnitine is another compound reported to protect skeletal muscle or tumor cells against simvastatin induced toxicity (Arduini et al., 2004; Costa et al., 2013). Figures 3A,B, respectively, show that 1 mM L-carnitine protected against inhibition of complex I respiration stimulated by ADP or FCCP.

### Increase in lactate production by 1 μM simvastatin was prevented by mevalonate, Co-Q10 or L-carnitine in skeletal muscle fibers

**Figure 4:**
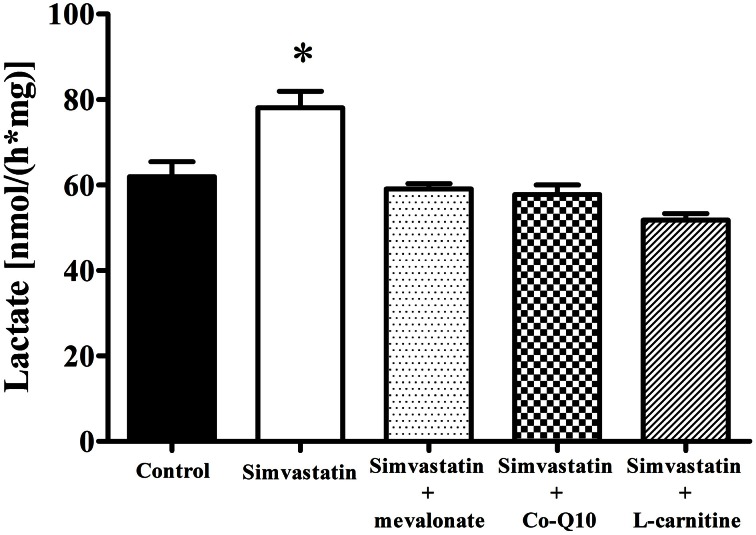
**Increase in lactate production by 1 μM simvastatin was prevented by mevalonate, Co-Q10 or L-carnitine in skeletal muscle.** An aliquot of MiR05 medium containing non-permeabilized skeletal muscle samples pre incubated by 1 h with 1 μM simvastatin or DMSO, or coincubated with 100 μM mevalonate, 10 μM coenzyme Q10 or 1 mM L-carnitine was added to a medium containing 50 mM hidroxilamine, 50 mM tris, 800 μM NAD^+^, 40 U lactate dehydrogenase, pH 10 at 25°C, for spectrofluorimetric assay of lactate release. ^*^p < 0.05 vs. control by one-way analysis of variance. *N* = at least 5 independent experiments.

Treatment of hypercholesterolemic patients with statins has been reported (De Pinieux et al., 1996) to increase lactate/pyruvate ratio in blood serum. In fact, this ratio can be used as a non-invasive test to detect impairment or toxic effects on mitochondrial energy linked metabolism (Robinson, 1989; Munnich et al., 1992; Chariot et al., 1994). Here we present data showing that 1 h incubation of skeletal muscle tissue with 1 μM simvastatin caused an increase of 26% in lactate production (Figure 4) that was abolished by the co-incubation of simvastatin with each of the compounds 100 μM mevalonate, 10 μM Co-Q10, or 1 mM L-carnitine.

### Decrease in coenzyme Q10 by 1 μM simvastatin was prevented by mevalonate but not by L-carnitine in skeletal muscle fibers

**Figure 5:**
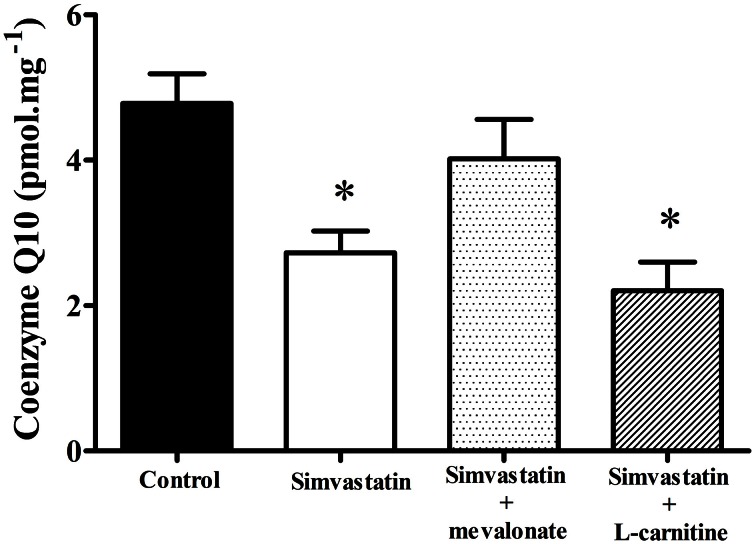
**Determination of coenzyme Q10 in skeletal muscle incubated with 1 μM simvastatin alone or in the presence of mevalonate or L-carnitine.** Permeabilized skeletal muscle samples were incubated in MiR05 medium in the presence of 1 μM simvastatin, or DMSO for 1 h in the presence of 100 μM mevalonate, 10 μM coenzyme Q10, or 1 mM L-carnitine. After tissue homogenization, extraction was performed utilizing perchloric acid and cold methanol. Coenzyme Q10 was measured with a Shimadzu HPLC. ^*^p < 0.05 vs. control by one-way analysis of variance. *N* = at least 9 independent experiments.

In order to ascertain the possible role of ubiquinone in the mechanism of statins induced mitochondrial dysfunction (Ghirlanda et al., 1993; Laaksonen et al., 1995; Thibault et al., 1996; Miyake et al., 1999; Rundek et al., 2004; Paiva et al., 2005; Littarru and Tiano, 2010) we assayed the Co-Q10 content in permeabilized skeletal muscle tissues after 1 h incubation with 1 μM simvastatin. Figure 5 shows that under the same experimental conditions in which the statin promotes inhibition of respiration the reduced levels of Co-Q10 were about 40% decreased relative to the control experiment. Interestingly, 100 μM mevalonate but not 1 mM L-carnitine prevented the decrease in Co-Q10 content. Therefore, we hypothesized that either L-carnitine or Co-Q10 protected against simvastatin-induced inhibition of respiration by acting as antioxidants. Accordingly, this inhibition of respiration was not mediated by the ability of Co-Q10 to transfer electrons from complex I and II to complex III, but rather by the action of Co-Q10 as a free radical scavenger. In order to ascertain this possibility we next investigated the effects of simvastatin alone or in co-incubation with 100 μM mevalonate, 10 μM Co-Q10, or 1 mM L-carnitine on hydrogen peroxide production by skeletal muscle fibers mitochondria.

### Increase in hydrogen peroxide by 1 μM simvastatin was prevented by mevalonate, Co-Q10 or L-carnitine in skeletal muscle fibers

**Figure 6:**
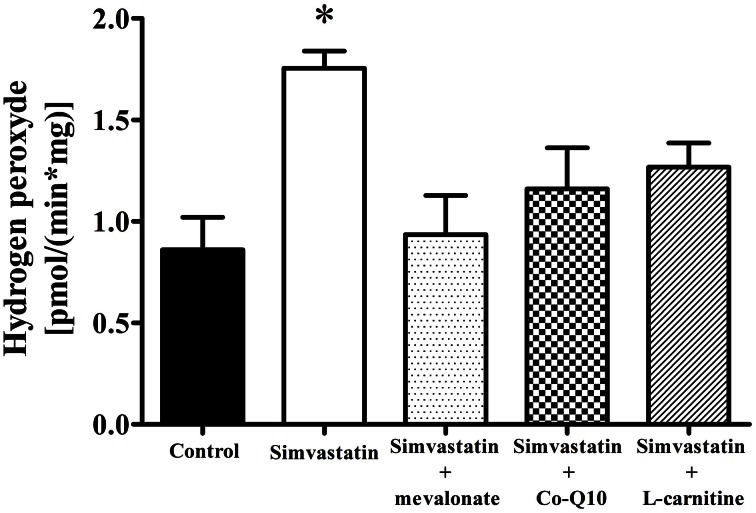
**Increase in hydrogen peroxide release by 1 μM simvastatin was prevented by mevalonate, Co-Q10 or L-carnitine in skeletal muscle.** Permeabilized skeletal muscle samples were incubated, for 1 h, in medium MiR05 containing 1 μM simvastatin or DMSO, 10 μM Amplex red, 1 U/mL horseradish peroxidase, 10 mM glutamate, 5 mM malate, or simultaneous incubated with 100 μM mevalonate, 10 μM coenzyme Q10 or 1 mM L-carnitine. Changes in fluorescence were monitored on a spectrofluorometer. ^*^p < 0.05 vs. control by one-way analysis of variance. *N* = at least 5 independent experiments.

Literature data also report that statin treatment may increase the rates of superoxide and hydrogen peroxide production in muscular cells (Kwak et al., 2012) and in muscle tissues from patients (Bouitbir et al., 2012b). In this study we assayed hydrogen peroxide and found that 1 h incubation of skeletal muscle tissues with 1 μM simvastatin promoted an increase of about 103% in the rate of hydrogen peroxide production compared to the controls. Similarly to what happens with respiration and lactate production, this increase in H_2_O_2_ was abolished by co-incubation of simvastatin with 100 μM mevalonate, 10 μM Co-Q10, or 1 mM L-carnitine (Figure 6).

## Discussion

The present work provides evidence that 1 h incubation of skeletal muscle fibers with 1 μM simvastatin promotes mitochondrial dysfunction associated with inhibition of respiration, decrease in the content of Co-Q10 and increased rates of hydrogen peroxide production.

Most literature data on statins toxicity was obtained from *in vivo* or *in vitro* experiments using different animal models and higher doses than those found in the serum of hypercholesterolemic patients (Sacher et al., 2005; Sirvent et al., 2005a; Kaufmann et al., 2006; Nadanaciva et al., 2007). Here we analyzed the mechanisms underlying mitochondrial dysfunctions associated with the lowest simvastatin concentration (1 μM) that significantly inhibited respiration supported by complex I and II linked substrates, under our experimental conditions. Although the assays were made *in vitro* using muscle biopsies, the present findings may be relevant to the understanding of statins toxicity *in vivo* that produces a variety of myophatic symptoms including myalgia, muscle cramps, and rarely rhabdomyolysis. These symptoms are more frequent in patients treated with high statin doses (80 mg/daily) and are increased by factors or conditions that increase serum and muscle statin concentrations (grape fruit juice, hypothyroidism, diabetes mellitus, advanced age, liver, and kidney diseases), and factors that increase muscle susceptibility to injury such as alcohol consumption, drug abuse, heavy exercise, and some muscle diseases (Venero and Thompson, 2009).

In the present study we did not find significant difference in resting respiration between statin treatment and controls. In contrast, a significant inhibition of both phosphorylating and uncoupled respiration, relative to the controls (Figure 1) suggests that mitochondrial number and density were normal and that the maximal respiratory capacity of skeletal muscle was decreased by simvastatin. This interpretation is supported by the citrate synthase assay (Figure 2) and is in line with those by Kwak et al. (2012) using myotube cells treated with simvastatin.

Increase in lactate levels, decrease in ATP production, and stimulated glycolysis have also been associated with inhibition of muscle respiration by statins impairment of mitochondrial functions (Robinson, 1989; Munnich et al., 1992; Chariot et al., 1994). Here we show that both inhibition of respiration and increase in lactate production were prevented by co-incubation of simvastatin with L-carnitine, mevalonate or Co-Q10; compounds known to protect against statins toxicity (Arduini et al., 2004; Sacher et al., 2005; Kettawan et al., 2007; Oliveira et al., 2008; Costa et al., 2013). These results are consistent with literature data (Kaufmann et al., 2006; Sirvent et al., 2008) proposing that simvastatin-induced inhibition of oxygen consumption in skeletal muscle is mediated by inhibition of Co-Q10 synthesis. On these grounds, the present results suggest that L-carnitine might also be protecting against the decrease in Co-Q10.

Co-Q10 is a mobile carrier that collects electrons from complex I and II transferring them to complex III and, in addition, in its reduced form (ubiquinol) acts as a potent antioxidant (Graham et al., 2009; Deichmann et al., 2010; Figueira et al., 2013). Supplementation with Co-Q10 has demonstrated effectiveness in ameliorating neurodegenerative diseases, cerebellar ataxia, heart failure, and muscular symptoms (Kaikkonen et al., 2002; Naini et al., 2003; Mabuchi et al., 2007; Littarru and Tiano, 2010). Therefore, we assayed the Co-Q10 content in skeletal muscle tissue treated with 1 μM simvastatin. In agreement with literature data (Ghirlanda et al., 1993; Laaksonen et al., 1995; Thibault et al., 1996; Miyake et al., 1999; Rundek et al., 2004; Paiva et al., 2005; Littarru and Langsjoen, 2007) our results indicate that simvastatin significantly decreased the content of the reduced form of Co-Q10. Interestingly, the co-incubation with L-carnitine, in contrast to the co-incubation with mevalonate that also protects against simvastatin-induced inhibition of respiration, did not protect against Co-Q10 depletion. This indicates that the decreased content of Co-Q10 is limiting the rate of mitochondrial respiration in the presence of simvastatin mainly due to its property to remove or scavenging free radicals and not due to its property to transfer electrons from complexes I and II to complex III. As a matter of fact, Panov et al. (2005) provided evidence that inhibition of respiration by superoxide, at the levels of complex I and complex II, may result from damage to 4Fe-4S clusters. Indeed, complex I has six and complex II has one 4Fe-4S clusters rendering these structures highly sensitive to the damaging effects of superoxide. Accordingly, previous data indicate that enzymes containing 4Fe-4S clusters are particularly vulnerable to damaging by superoxide or peroxinitrite radicals (Flint et al., 1993; Radi et al., 1994; Fridovich, 1995; Bouton et al., 1996; Panov et al., 2005; Demicheli et al., 2007).

L-carnitine is known to protect against mitochondrial dysfunctions associated with oxidative stress caused by a series of conditions such as aging, ischemia reperfusion, inflammation, degenerative diseases, carcinogenesis and drug toxicity, *in vivo* or *in vitro* (Moretti et al., 2002; Binienda, 2003; Kumaran et al., 2004, 2005; Sener et al., 2004; Virmani et al., 2004; Binienda et al., 2005; Keil et al., 2006; Yapar et al., 2007; Shen et al., 2008; Silva-Adaya et al., 2008; Elinos-Calderon et al., 2009; Vamos et al., 2010; Ye et al., 2010; Zhang et al., 2010). Given the properties of L-carnitine to scavenge reactive oxygen (Gulcin, 2006; Mescka et al., 2011) and to bind Fe^2+^ (Gulcin, 2006), we propose that this molecule may directly interact with 4Fe-4S clusters protecting the respiratory complexes I and II against the attack by the superoxide radical. This proposition is not in contrast to the results reported by Benati et al. (2010) showing that simvastatin decreases the capacity of macrophages to phagocyte and kill bacteria by impairment of oxidative burst, a rather different mechanism not involving the mitochondrial respiratory chain.

In addition, it is noteworthy to remind that 60 μM simvastatin-induced MPT and cell necrosis were sensitive to L-carnitine or piracetam in a dose-dependent fashion and mediated by additive mechanisms (Costa et al., 2013). In the present work we observed that piracetam did not affect the inhibition of respiration or the increase in H_2_O_2_ production induced by 1 μM simvastatin (results not shown). These results suggest that in the previous work (Costa et al., 2013) the role of piracetam on the protection against high simvastatin concentrations (60 μM) was mediated by the ability of the compound to protect against Ca^2+^-induced alterations in membrane fluidity (Keil et al., 2006). This occurs via unspecific interactions of piracetam with the polar head groups of biological membranes (Keil et al., 2006). Indeed, Ca^2+^ binding to inner membrane cardiolipin causes important alterations in the lipid organization that favors the burst of mitochondrial ROS that triggers Ca^2+^-induced MPT (Grijalba et al., 1999).

## Conclusions

Considering that the three compounds (mevalonate, Co-Q10, or L-carnitine) that prevented the inhibition of respiration by simvastatin also protected against stimulation of hydrogen peroxide generation, we may conclude that reactive oxygen is the common denominator in the mechanism of respiration inhibition by statins. In addition, the lack of protection against Co-Q10 depletion by L-carnitine indicates that this compound as well as Co-Q10 acts directly as radical scavenger in the protection against simvastatin-induced oxidative damage to skeletal muscle mitochondria.

### Conflict of interest statement

The authors declare that the research was conducted in the absence of any commercial or financial relationships that could be construed as a potential conflict of interest.
